# Parenting Practices at 24 to 47 Months and IQ at Age 8: Effect-Measure Modification by Infant Temperament

**DOI:** 10.1371/journal.pone.0152452

**Published:** 2016-03-30

**Authors:** Shiau Yun Chong, Catherine R. Chittleborough, Tess Gregory, Murthy N. Mittinty, John W. Lynch, Lisa G. Smithers

**Affiliations:** 1 School of Population Health, University of Adelaide, Adelaide, Australia; 2 Telethon Kids Institute, The University of Western Australia, Perth, Australia; 3 School of Social and Community Medicine, University of Bristol, Bristol, United Kingdom; The University of Queensland, AUSTRALIA

## Abstract

Cognitive development might be influenced by parenting practices and child temperament. We examined whether the associations between parental warmth, control and intelligence quotient (IQ) may be heightened among children in difficult temperament. Participants were from the Avon Longitudinal Study of Parents and Children (n = 7,044). Temperament at 6 months was measured using the Revised Infant Temperament Questionnaire and classified into ‘easy’ and ‘difficult’. Parental warmth and control was measured at 24 to 47 months and both were classified into 2 groups using latent class analyses. IQ was measured at 8 years using the Wechsler Intelligence Scale for Children and dichotomized (<85 and ≥85) for analyzing effect-measure modification by temperament. Linear regression adjusted for multiple confounders and temperament showed lower parental warmth was weakly associated with lower IQ score [β = -0.52 (95% CI 1.26, 0.21)], and higher parental control was associated with lower IQ score [β = -2.21 (-2.95, -1.48)]. Stratification by temperament showed no increased risk of having low IQ in temperamentally difficult children [risk ratio (RR) = 0.97 95% CI 0.65, 1.45)] but an increased risk among temperamentally easy children (RR = 1.12 95% CI 0.95, 1.32) when parental warmth was low. There was also no increased risk of having low IQ in temperamentally difficult children (RR = 1.02 95% CI 0.69, 1.53) but there was an increased risk among temperamentally easy children (RR = 1.30 95% CI 1.11, 1.53) when parental control was high. For both parental warmth and control, there was some evidence of negative effect-measure modification by temperament on the risk-difference scale and the risk-ratio scale. It may be more appropriate to provide parenting interventions as a universal program rather than targeting children with difficult temperament.

## Introduction

Cognitive ability is an important aspect of healthy child development. Intelligence quotient (IQ), derived from intelligence tests, is a marker of future health, academic achievement, occupational outcomes, and social development [1–3]. At the population level, increases in average IQ are associated with improvements in economic growth [4]. Children with lower IQ have increased risk of mortality and morbidity as well as lower occupational status and earnings in adulthood [5]. Cognitive abilities develop in early life through social interactions [6], and a nurturing environment is particularly important to facilitate cognitive development [7]. For instance, children with supportive parents who engage in learning activities are more ready to learn and develop their cognitive abilities.

Parental warmth and control are two aspects of parenting that are important for children’s development. There is some evidence that parental warmth is associated with children’s cognitive development [8,9]. Parents who understand the child’s needs may also be more likely to provide support to assist the child in developing learning skills such as mastery, security, autonomy, and self-efficacy [10]. On the other hand, low warmth parenting that uses verbal and physical punishments may hinder the child’s cognitive development. High parental control is characterized by behaviors that involve the use of pressure, solving problems for children, and making decisions for the child based on a parental perspective [11,12]. High parental control may be associated with lower levels of intrinsic motivation as children with controlling parenting are less likely to engage in activities and attempts that help them to learn [13]. Children of more controlling parents may also have poorer self-regulation [12], hence affecting their cognitive development.

Besides parenting practices, individual factors such as the child’s temperament also play a role in their development. Children who are high in approach to unfamiliar persons or objects, more adaptable to new environments and more positive in mood are linked to have higher IQ [14,15]. There is also some evidence that self-regulation is associated with IQ [16,17]. Self-regulation, which is the ability to consciously control activity, emotion, and attention, is an emerging component of temperament that is observable from the age of two [18,19]. Studies categorize temperament by combining traits including rhythmicity, approach, adaptability, intensity, and mood to create a construct of difficult temperament have found mixed results. One study reported no association between temperament and IQ [20], whereas another found that IQ was higher in children with difficult temperament than children with easy temperament [21]. Further studies are needed to determine the influence of temperament on children’s IQ.

This study aims to examine whether the associations between parenting warmth, control and IQ differ among children with different temperaments, *i*.*e*. the possibility that temperament is an *effect modifier* of the association between parental warmth and control on IQ. Our hypothesis that temperament would modify the associations between parenting warmth, control and IQ is based on the following rationale. Temperament is the individual differences in styles that are observable from early childhood [22]. In this study we use the Revised Infant Temperament Questionnaire (RITQ) measured at 6 months of age, and dichotomized into difficult versus easy or other categories as has been recommended [23]. It is possible that some temperament characteristics may help buffer children from adverse effects of negative parenting practices. For instance, children who are temperamentally easy are more capable at controlling their own emotions and more adaptable to their environment. Therefore, they are better in finding ways to fit in into their environmental context and less likely to be affected by parenting practices than temperamentally difficult children who have problems controlling their emotions and are less adaptable to their environment. If this hypothesis is true, we would expect that, given similar circumstances of growing up in a less positive parenting environment, children who are temperamentally easy would have fewer adverse outcomes compared to children with more difficult temperament. Understanding the associations between parenting practices (warmth and control) on children with different temperaments will help to determine whether parenting interventions should be targeted to children with specific temperaments or to all children.

In this current study, we are interested in effect-measure modification by temperament because our interest is to intervene on parenting practices (warmth and control) rather than child temperament. We focus on parenting practices because of the preponderance of interventions currently being used that are designed to improve parenting [24,25]. Interventions on temperament of infants and young children are typically through improving parent-child social relationships, and reinforcement activities delivered by parents or teachers [26]. Although parenting and temperament are measured at different time points (temperament at 6 months, parenting at 24 to 47 months), this study is interested in ‘effect-measure modification’ (whether the association of warmth, control, and IQ differs in children with different temperament), but not ‘mediation’ (examining the direct effect of temperament on IQ, and the indirect effect of temperament on IQ that goes through parenting). Therefore, the hypothesis put forward in this paper is an issue of effect-measure modification. In practice, the distinctions between effect-measure modification and interaction are often ignored. Traditionally, effect-measure modification and interaction are tested in regression analyses by including a product term. However, using regression models, it is not clear whether the coefficient of the interaction term should be interpreted as effect-measure modification or interaction, or both, or neither [27]. Interaction is widely used when interpreting findings that should be interpreted as effect-measure modification [27]. The distinctions between effect-measure modification and interaction are important especially when considering potential intervention and policy recommendations [27,28]. For public health intervention purposes, only one intervention is considered in effect-measure modification, *i*.*e*. the main exposure, while in interaction, potential intervention on both exposures is considered. Conceptually, effect-measure modification occurs when the effect of the main exposure (parenting) on an outcome (IQ) differs across strata of a second exposure (temperament) [27]. Effect-measure modification on the risk-difference scale can be written as:

$$E\left[Y_{P_{1}}|T=t_{1},C=c\right]-E\left[Y_{P_{0}}|T=t_{1},C=c\right]\neq E[Y_{P_{1}}|T=t_{0},C=c]-E[Y_{P_{0}}|T=t_{0},C=c]$$ (1)

where *Y* denotes the outcome under study, *T* denotes the effect modifier, *P* denotes the exposure of interest, and *C* denotes a set of confounders. Eq 1 is read as the expectation of the difference in outcome (*Y*) between low warmth parenting (*P*_*1*_) and high warmth parenting (*P*_*0*_) in stratum *T*_*1*_ of effect modifier (conditioned on *C*) is not equal to the expectation of the difference in outcome (*Y*) between low warmth parenting (*P*_*1*_) and high warmth parenting (*P*_*0*_) in stratum *T*_*0*_ (conditioned on *C*). In effect-measure modification (Eq 1), the relationship between *P* and *T* are asymmetric, *i*.*e*. only the effect of *P* on *Y* is of interest, *T* only concerns whether the effect of *P* on *Y* differ across different value of *T*. Interaction is different from effect-measure modification in that it concerns whether the joint effect of the two exposures differs from the combined independent effects. Interaction on the risk-difference scale can be written as:

$$E[Y_{P_{1}T_{1}}|C=c]-E[Y_{P_{0}T_{1}}|C=c]\neq E[Y_{P_{1}T_{0}}|C=c]-E[Y_{P_{0}T_{0}}|C=c]$$ (2)

In interaction (Eq 2), the role of *P* and *T* are symmetric, *i*.*e*. both *P* and *T* have causal effects on *Y*. Given that our interest is to intervene on *P* (parenting) but not on *T* (temperament), it is essential to understand the effect of *P* on *Y* rather than the joint effect of *P* and *T* on *Y*, therefore effect-measure modification is of interest in this current study rather than interaction.

The presence or absence and the magnitude of effect-measure modification depend on which scale the association is measured—risk-difference or risk-ratio scale. The risk-difference scale estimates the extent to which the effect of the two exposures, *i*.*e*. parenting and temperament operating together exceeds the effect of each added together [27]. The risk-difference scale helps us to identify target groups as it allows us to see the absolute gain in outcome if an intervention is targeted at certain subgroup, which can help making public policy decisions when resources are finite (for example, see Appendix A in S1 File). On the other hand, the risk-ratio scale estimates the extent to which the effect of both exposures operating together exceeds the product of the effects of the two exposures. It is unclear how to interpret effect-measure modification on the risk-ratio scale, as it does not allow us to determine which subgroup to treat, but it is thought to be useful for investigation of possible biological pathways [29]. While there is consensus that the risk-difference scale is considered more important for public policy action and interventions, the risk-difference scale is often not reported in many studies [30]. Even though effect-measure modification is widely studied in epidemiological research, most studies have not provided enough information about the size and statistical significance of the effect-measure modification on both the risk-difference and risk-ratio scale [27,30].

This study examined the *effect-measure modification* of the association between parental practices (warmth and control) and IQ by child temperament. We reported the effect-measure modification on both the risk-difference and risk-ratio scale for transparency and completeness [31,32], however, results on the risk-difference scale are more pertinent to this research question because of the implications for public health intervention.

## Methods

### Study design

**Fig 1:**
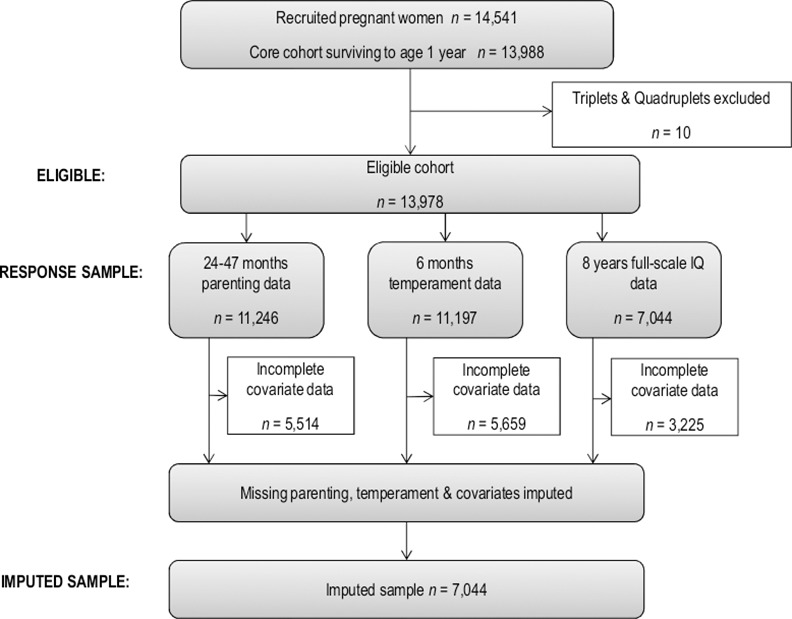
Eligible cohort and numbers included.

The Avon Longitudinal Study of Parents and Children (ALSPAC) is a population-based prospective study investigating the influence of genetic and environmental characteristics on health and development in parents and children. A total of 14,541 pregnant women who resided in the Southwest of England with expected delivery date between 1^st^ April 1991 and 31^st^ December 1992 were recruited, and this includes 72% of the eligible mothers [33]. The ALSPAC sample is broadly representative of the population living in Avon and the whole of Britain at the time although ethnic minorities and unmarried couples were slightly underrepresented [33]. Follow up assessments have been administered frequently through questionnaires and clinical assessments. The length of follow-up and the breadth of data collection provide valuable data that can be used as confounders. The ALSPAC sample consists of 13,988 infants who were alive at one year (Fig 1). The study website contains details of all the data that is available through a fully searchable data dictionary (http://www.bris.ac.uk/alspac/researchers/data-access/data-dictionary/).

### Ethics statement

Ethical approvals were obtained from the Human Research Ethics Committee, University of Adelaide, ALSPAC Law and Ethics committee and the four Local Research Ethics committees: Southmead, Frenchay, Bristol and Weston health authorities. Written consent was obtained from the original participants and from the parents, next of kin, caretakers, or guardians on behalf of the children enrolled in ALSPAC.

### Measures

#### IQ (the outcome)

IQ was assessed using the Wechsler Intelligence Scale for Children (WISC-III ^UK^) when the children were 8 years of age [34]. Children’s scores on five verbal and five performance subtests were summarized into domains of verbal IQ and performance IQ, which were then combined to yield the full-scale IQ. All tests were administered by the ALSPAC psychology team. To reduce the length of the session, alternate items were used for all subtests, except the coding subtest which was administered in full. Individual items within each subtest were summed and multiplied by 2 for picture completion, information, arithmetic, vocabulary, comprehension, and picture arrangement; multiplied by 5/3 for similarities; and multiplied by 3/2 for object assembly and block design. This made the raw scores comparable to those that would have been achieved had the full test been administered. Raw scores were converted to age-scaled scores according to standard procedures [34]. The IQ scores were standardized on a normal British population in the early 1990s to have a mean of 100 and standard deviation of 15.

IQ was used as a continuous variable in regressions but we used dichotomized outcome when analyzing effect-measure modification for ease of interpretation of results [32]. IQ was dichotomized at <85 (‘low IQ’) and ≥85 (‘normal to high IQ’). Although other IQ cut-points were tested and results found to be similar (Appendix D in S1 File), the cut-point at IQ 85 reflected a balance between having an IQ score at one SD below the mean and an adequate sample in each cell for effect-measure modification analysis. Furthermore, this group of children have lower human capital and greater likelihood of needing supportive resources [35]. Of the participants for whom full-scale IQ score was available, 12.0% (*n* = 847) were classified as having a low IQ score.

#### Parenting warmth and control (the exposures of interest)

Parenting warmth and control were reported by mothers using items selected from ALSPAC questionnaires. There were eight items for warmth and nine items for control across 24 to 47 months (see Table 1). The parenting measures correspond to warmth and control which are identified as important aspects of parenting [36]. In order to encourage continued participation of ALSPAC members, parenting questions were phrased in a way that minimized offence. Mothers answered the questions with 3- to 5-point Likert scales that ranged from ‘never’ to ‘frequently’.

**Table 1:** Parenting measures in the ALSPAC questionnaires.

Parenting questions	Age	Responses
*Parental warmth*		
Mother smacks child during tantrums	42 months	Never, rarely, once a month, once a week, daily
Mother shouts at child when naughty	24, 42 months	Every day, several times a week, once a week, rarely, never
Child is slapped	24 months	Every day, several times a week, once a week, rarely, never
Child is kissed or cuddled	24, 38, 42 months	Nearly every day, 3–5 times per week, 1–2 times per week, < once per week, never
Child is praised	24 months	Every day, several times a week, once a week, rarely, never
*Parental control*		
Mother reasons with child during tantrums	30, 42 months	Often, sometimes, never
Child has some choice at meals	30, 42 months	Free choice, select choice, no choice
Child has some choice with clothes	30, 42 months	Free choice, select choice, no choice
Parent and child have battle of wills	30, 42, 47 months	Never, rarely, sometimes, frequently

We used latent class analysis (LCA) in SAS statistical software version 9.2 (SAS Institute, Inc, Cary, North Carolina) to investigate the patterns of parenting in our study population. LCA identifies groups of parents with distinct characteristics based on their responses to the parenting items. LCA is a model-based cluster analysis that provides a set of class-assignment probabilities for each respondent [37–39]. The latent classes were created based on two parameters: the latent class membership probabilities and the conditional item-response probabilities [38]. The latent class membership probabilities estimate the proportion of the population that fall into a given class. The conditional item-response probabilities are probabilities of responding to each question, given the class membership. The item-response probabilities are conceptually similar to factor loadings in factor analysis. However, they are probabilities rather than coefficients [40]. Individual posterior probabilities of membership in each latent class were obtained by applying Bayes’ theorem [38]. Models with two-, three-, and four-class solutions were tested and the best model was chosen based on the log-likelihood, Bayesian Information Criterion, and the face validity of the classes [37]. A two-class solution was chosen for both warmth and control. Each respondent was assigned a class based on their highest probability of membership. The classes were given descriptive labels based on consensus of the authors after reviews of each class’s characteristics.

#### Temperament (the potential effect-modifier)

Temperament was assessed when the infants were 6 months old using an adaptation of the RITQ (S2 File)[41]. RITQ is a valid and reliable measure of temperament [18,42]. The internal consistency of the adapted RITQ is consistent with the internal consistencies of the original questions included in the RITQ [43]. Scores for each subscale were derived according to the procedure introduced in the test manual [41].

Infant temperament was classified into two groups based on the scores on five temperament subscales (rhythmicity, approach, adaptability, intensity and mood) [44]. Infants were defined as difficult if they scored greater than the mean on at least four of the five subscales and greater than one standard deviation above the mean on at least two subscales. Infants who did not satisfy the difficult definition were classified into a single group called ‘easy or other’. The classification of infant temperament utilized ALSPAC-specific norms, rather than norms from the test manual, which we have previously demonstrated may reduce misclassification of temperament [43]. There were 1655 (15%) of the infants with temperament data available who were classified as difficult.

#### Confounding

Confounders were determined *a priori* [45] based on factors that might confound the association between parenting and IQ. Confounders included parent level factors such as indicators of socio-economic position, parents’ physical and mental health and child level factors such as intrauterine growth (see Table 2 for full list).

**Table 2:** IQ, parenting, temperament, and demographic characteristics of response, complete case and imputed sample.

	Response sample		Complete data sample (n = 3,665)	Imputed sample (n = 7,044)
	n	Mean (SD) or %	Mean (SD) or %	Mean (SD) or %
**Total IQ, 8 years**	7,044	104.2 (16.5)	106.3 (16.4)	104.2 (16.5)
Low IQ (<85)	847	12.0	9.7	12.0
Normal to high IQ (≥85)	6,197	88.0	90.3	88.0
**Parenting, 24–47 months**				
Parental warmth				
High warmth	5,205	45.7	48.6	46.9
Low warmth	6,184	54.3	51.4	53.1
Parental control				
Less controlling	6,522	58.0	59.1	59.4
High controlling	4,724	42.0	40.9	40.6
**Temperament, 6 months**				
Easy or other	4,169	85.1	86.1	84.6
Difficult	1,655	14.9	13.9	15.4
**Child covariables**				
Sex, female	13,976	48.3	49.3	50.0
Birth weight (grams)	13,798	3392.0 (559.3)	3446.2 (516.7)	3414.6 (554.2)
Gestational age (weeks)	13,976	39.4 (1.9)	39.5 (1.6)	39.4 (1.9)
Ethnicity, non-white	12,083	5.0	2.1	4.1
**Parent covariates**				
Maternal age (years)	13,978	28.0 (5.0)	29.2 (4.3)	28.0 (5.0)
Maternal smoking in first 3 months pregnancy	13,158	25.0	14.4	18.3
Maternal alcohol consumption in first 3 months pregnancy				
Never	5,917	45.5	44.3	44.1
Less than 1 glass per week	5,034	38.7	41.3	40.7
One or more glass per week	1,804	13.9	13.1	13.3
One or more glass per day	250	1.9	1.3	1.8
No partner / not living with partner	13,179	8.7	1.9	5.6
Home ownership, rented/other	13,027	26.6	11.7	16.9
Household crowding, >1 person/room	12,084	6.9	2.6	4.1
Maternal highest education				
None / CSE / vocational	3,728	26.7	16.8	22.2
O level	4,296	30.7	35.1	34.9
A level	2,794	20.0	28.6	26.7
Degree or higher	1,600	11.5	19.6	16.2
Partner’s highest education				
None / CSE / vocational	4,124	34.5	21.2	28.9
O level	2,540	21.3	22.1	21.8
A level	3,105	26.0	30.1	27.8
Degree or higher	2,171	18.2	26.6	21.5
Parental highest social class				
Professional / managerial (I/II)	6,342	55.1	66.3	60.0
Skilled manual / non-manual (III)	4,481	38.9	30.9	35.5
Unskilled / semiskilled manual (IV/V)	682	5.9	2.8	4.5
Financial difficulties	12,086	10.0	5.7	8.1
Social support				
Low	4,142	38.1	32.3	36.1
Medium	3,736	34.4	36.8	35.6
High	2,999	27.6	30.9	28.3
Maternal depression	10,929	21.9	14.8	17.9
Partner’s depression	7,605	7.9	5.5	7.7
Maternal health, often unwell / hardly ever well	11,317	5.5	4.9	5.1

*CSE* Certificate of Secondary Education, *IQ* Intelligence Quotient.

Measures of socio-economic position were obtained from mothers’ self-reported questionnaires from pregnancy until 8 weeks postpartum. Mother’s and partner’s education were categorized into 4 levels consistent with the UK education system: none or certificate of secondary education (CSE) or vocational; O level; A level; degree. Parents’ social class was based on the highest occupation of either parent, and categorized using standard UK classifications of occupation, ranging from class I (highest), II, III-non-manual, III-manual, IV, and V (lowest). Mothers’ financial difficulties in affording food, clothing, heating, rent or mortgage was assessed at 32 weeks gestation with possible responses ranging from 1 (very difficult) to 4 (not difficult). The sum of the scores of each of the 5 items was subtracted from 20 to derive a total financial difficulties score. A total score of 0 represented no financial difficulties and 15 represented maximum financial difficulties. Mothers with a score of 9 and above were defined as experiencing financial difficulties and this included approximately 10% of the cohort [46]. Household crowding was categorized according to whether there were ≤ 1 or > 1 person per room. Home ownership was categorized into owned or mortgaged and rented or other. Mother’s social support was measured using a set of 10 items specifically designed for the cohort. These items represented statements in relation to financial, emotional and instrumental support the mothers received from their partners, friends, families and official agencies. Scores were summed from 0 (lowest) to 30 (highest). The social support score was separated into three groups of equal size (‘low’, ‘medium’, and ‘high’). Mother’s marital status was classified as ‘married or cohabiting’ and ‘not married or not living with partner’.

At 8 months postpartum, mothers self-rated their own health as always well, mostly well, often feel unwell, or hardly ever well. Mothers’ and partners’ depression was assessed using the ten items from the Edinburgh Postnatal Depression Scale (EPDS) at 18 weeks antenatal and 8 weeks postpartum. The EPDS is validated for use among parent groups during the postnatal and pregnancy period [47,48]. Mothers and partners with a score of ≥12 in either measurement time were considered as displaying depressive symptoms [47].

Mothers were asked whether or not they smoked during the first three months of pregnancy. Mothers’ alcohol consumption during the first three months of pregnancy was classified as never, < 1 glass per week, and ≥ 1 glasses per week or ≥ 1 glasses per day. Child’s gestational age and birth weight were collected by ALSPAC staff from obstetric data.

### Analysis

Correlations between parenting, temperament, IQ and all confounders are included in Appendix B in S1 file. A series of analyses were undertaken to examine the effect of the parenting dimensions (warmth, control) on children’s full-scale IQ (continuous score) using linear regression modelling. In the first step, univariable associations between parenting warmth on IQ and parenting control on IQ were examined separately (Model 1). We then adjusted for temperament (Model 2). In Model 3, parenting warmth model was adjusted for parenting control and all other confounding variables described above, parenting control model was adjusted for parenting warmth and all other confounding variables.

We estimated effect-measure modification on both risk-difference and risk-ratio scales as outlined by Knol and VanderWeele [32]. Using dichotomized IQ, Poisson regressions were used to estimate risk ratio (RR) estimates for each stratum of parenting (P) and temperament (T): i) high warmth parenting and easy temperament (${\text{RR}}_{{\text{P}}_{0}{\text{T}}_{0}}$)(reference category); ii) high warmth parenting and difficult temperament $\left({\text{RR}}_{{\text{P}}_{0}{\text{T}}_{1}}\right)$; iii) low warmth parenting and easy temperament $\left({\text{RR}}_{{\text{P}}_{1}{\text{T}}_{0}}\right)$; and iv) low warmth parenting and difficult temperament $\left({\text{RR}}_{{\text{P}}_{1}{\text{T}}_{1}}\right)$. Next, the RR for parenting within strata of temperament was estimated. Poisson models with robust errors were used to estimate RR due to convergence problems with log-binomial models.

A relative excess risk due to interaction (RERI) was calculated to give the measure of effect-measure modification on the risk-difference scale, and 95% CIs were obtained by the delta method [49].

$$RERI={RR}_{P_{1}T_{1}}-{RR}_{P_{0}T_{1}}-{RR}_{P_{1}T_{0}}+{RR}_{P_{0}T_{0}}$$ (3)

RERI > 0 indicates the effect-measure modification is positive (the effect of the exposure and the effect modifier operating together is greater than the effect of each added together), RERI < 0 indicates the effect-measure modification is negative, RERI of 0 indicates there is no effect-measure modification on the risk-difference scale. Effect-measure modification on the risk-ratio scale is taken as:

$$Ratio\;of\;RRs=\frac{{RR}_{P_{1}T_{1}}\;X\;{RR}_{P_{0}T_{0}}}{{RR}_{P_{0}T_{1}}{X\;RR}_{{P_{1}T}_{0}}}$$ (4)

If the ratio of RRs >1, the effect-measure modification is positive (the effect of the exposure and the effect modifier operating together is greater than the product of the effect of the exposure and the effect modifier). A ratio of RRs < 1 indicates the effect-measure modification is negative. A ratio of RRs = 1 means the effect of both exposures together is equal to the product of the effect of the two exposures considered separately.

Analyses were performed using Stata version 13.0 (Stata Corp, College Station, Texas).

#### Multiple imputation for missing data

We used multiple imputation by chained equation to impute missing data. Imputed datasets were generated under the missing at random assumption that the probability of data being missing is dependent on the observed data [50]. Variables included in the imputation model were parenting warmth, control, temperament, all confounders, breastfeeding, HOME inventory, all three measures of IQ (full-scale IQ, verbal IQ, and performance IQ) and interaction terms between parenting and temperament. Breastfeeding and HOME inventory variables were two auxiliary variables that were added to the imputation model because they are related to the outcome (IQ) and may enhance the prediction of missing values. Fifty cycles of regression switching were undertaken and 20 imputed datasets were generated. We used the multiple imputation then deletion technique [51] where analyses were conducted on respondents only with non-imputed outcome data. All analyses were performed on imputed data (n = 7,044).

## Results

Table 2 shows the socio-demographic characteristics of ALSPAC response sample, respondents who had complete data on IQ, parenting, temperament, and all covariables (complete case, *n* = 3,665), and the imputed sample (*n* = 7,044). Participants with complete data had a higher proportion of mothers with higher warmth and lower control parenting, higher education, and higher social class, and lower proportions of mothers with financial difficulties, who smoked during pregnancy and were unmarried or not living with partner. Lower proportions of children in the complete case sample were non-white, and had low IQ.

Table 3 shows the associations between parenting and IQ at 8 years (*n* = 7,044) using linear regressions. Models 1 and 2 provide some evidence that low parental warmth and high parental control were associated with lower IQ at 8 years. In the fully-adjusted model (Model 3), children experiencing low warmth parenting had 0.52 (95% CI -1.26, 0.21) point lower IQ than children experiencing high warmth parenting. Children whose parents demonstrated high control had 2.21 (95% CI: -2.95,-1.48) point lower IQ than children whose parents demonstrated low control. The association between difficult temperament and IQ was negligible (β = -0.12, 95% CI -1.13, 0.90).

**Table 3:** Association between parenting warmth and control, and child temperament on children’s IQ (Imputed sample, n = 7,044).

	Model 1			Model 2			Model 3		
	β	95% CI	*p*	β	95% CI	*p*	β	95% CI	*p*
Warmth									
High	Ref			Ref			Ref		
Low	-3.21	-3.99, -2.42	<0.001	-3.04	-3.84, -2.24	<0.001	-0.52	-1.26, 0.21	0.166
Temperament									
Easy or Other				Ref			Ref		
Difficult				-0.14	-1.27,1.00	0.976	-0.12	-1.13, 0.90	0.824
Control									
Less controlling	Ref			Ref			Ref		
High controlling	-3.33	-4.13, -2.53	<0.001	-3.29	-4.11, -2.47	<0.001	-2.21	-2.95, -1.48	<0.001
Temperament									
Easy or Other				Ref			Ref		
Difficult				-0.25	-1.49, 0.97	0.683	-0.12	-1.13, 0.90	0.824

Model 1 is unadjusted. Model 2 is adjusted for temperament. In Model 3, parenting warmth is adjusted for temperament and all the covariables (maternal smoking, alcohol consumption, birth weight, gestation at birth, sex, ethnicity, maternal age, partner status, financial difficulties, maternal and partner’s education, parental social class, home ownership, household crowding, maternal health, social support, maternal and partner’s depression), parenting control is adjusted for parenting warmth and all the covariables.

Table 4 shows effect-measure modification of the association between parenting warmth and IQ by temperament. Among temperamentally easy children, low warmth parenting was associated with 12% higher risk of low IQ (95% CI 0.95, 1.32), whereas in the stratum of temperamentally difficult children, low warmth parenting did not increase the risk of low IQ (RR 0.97 95% CI 0.65, 1.45). Compared with the reference category of children who had high warmth parenting and easy or other temperament, children with low warmth parenting or difficult temperament, or both, had about 12 to 17% increased risk of having low IQ. The RERI of -0.19 (95% CI -0.65, 0.27) indicated small negative effect-measure modification by temperament on the risk-difference scale, *i*.*e*. the combined risks of both low warmth parenting and difficult temperament (RR 1.12) was lower than expected (RR 1.31) when based on summing the individual risks of low warmth parenting (RR 1.14) and difficult temperament (RR 1.17) Similarly, for effect-measure modification on the risk-ratio scale, the ratio of RRs was 0.84 (95% CI 0.56, 1.25) indicating that the combined risks of both low warmth parenting and difficult temperament (RR 1.12) was lower than expected (RR 1.33) when based on multiplying the individual risks of low warmth parenting and difficult temperament.

**Table 4:** Effect-measure modification of the effect of parenting warmth on IQ (<85) by child temperament (Imputed sample, n = 7,044)

	High warmth parenting		Low warmth parenting		RR (95% CI) for low warmth parenting within strata of temperament type
	N Low IQ/High IQ	RR (95% CI)	N Low IQ/High IQ	RR (95% CI)	
Easy or other temperament	283/2565	1.00 (Ref)	433/2749	1.14 (0.97, 1.34), p = 0.106	1.12 (0.95, 1.32), p = 0.182
Difficult temperament	47/391	1.17 (0.85, 1.61), p = 0.327	84/492	1.12 (0.86, 1.45), p = 0.393	0.97 (0.65, 1.45), p = 0.872

Effect-measure modification on the risk-difference scale: RERI = -0.19 (-0.65, 0.27), p = 0.413. [RERI = 1.12–1.14–1.17+1.00 = -0.19 when estimated from the table]Effect-measure modification on the risk-ratio scale: Ratio of RRs = 0.84 (0.56, 1.25), p = 0.385. [Ratio of RRs = 1.12/(1.14 x 1.17) = 0.84 when estimated from the table]. RRs are adjusted for parenting control, maternal smoking, alcohol consumption, birth weight, gestation at birth, sex, ethnicity, maternal age, partner status, financial difficulties, maternal and partner’s education, parental social class, home ownership, household crowding, maternal health, social support, maternal and partner’s depression.

Table 5 shows the effect-measure modification of the association between parental control and IQ by child temperament. The increased risk of low IQ associated with high control parenting was 30% in easy or other temperament. There was no increased risk of having low IQ in difficult temperament children (RR 1.02 95% CI 0.69, 1.53) but the confidence intervals were wide in the stratum of temperamentally difficult children due to smaller numbers. Compared with children who had easy or other temperament and less controlling parenting, children with easy or other temperament and high control parenting had a 31% increased risk of having low IQ, children with difficult temperament and less controlling parenting and children with both difficult temperament and high control parenting had a 18% increased risk of having low IQ.

**Table 5:** Effect-measure modification of the effect of parenting control on IQ (< 85) by child temperament (Imputed sample, n = 7,044).

	Less controlling parenting		High controlling parenting		RR (95% CI) for high control parenting within strata of temperament type
	N Low IQ/High IQ	RR (95% CI)	N Low IQ/High IQ	RR (95% CI)	
Easy or other temperament	349/3195	1.00 (Ref)	367/2119	1.31 (1.12, 1.53), p = 0.001	1.30 (1.11, 1.53), p = 0.001
Difficult temperament	77/551	1.18 (0.90, 1.53), p = 0.228	54/332	1.18 (0.89, 1.59), p = 0.261	1.02 (0.69, 1.53), p = 0.907

Effect-measure modification on the risk-difference scale: RERI = -0.30 (-0.78, 0.18), p = 0.226. [Due to rounding, RERI = 1.18–1.31–1.18+1.00 = -0.31 when estimated from the table]. Effect-measure modification on the risk-ratio scale: Ratio of RRs = 0.77 (0.52, 1.15), p = 0.204. [Due to rounding, ratio of RRs = 1.18/(1.31 x 1.18) = 0.76 when estimated from the table]. RRs are adjusted for parenting warmth, maternal smoking, alcohol consumption, birth weight, gestation at birth, sex, ethnicity, maternal age, partner status, financial difficulties, maternal and partner’s education, parental social class, home ownership, household crowding, maternal health, social support, maternal and partner’s depression.

For parental control, the RERI of -0.30 (95% CI -0.78, 0.18) suggested a small negative effect modification, although confidence intervals were wide. The measure of effect-measure modification on the risk-ratio scale was 0.77 (95% CI 0.52, 1.15) also indicated a small negative effect-measure modification.

Graphical illustrations of the effects of parenting warmth and control across stratum of temperament are included in Appendix C in S1 File. Other IQ cut points (80 and 90) were also tested and the results were similar (Appendix D in S1 File).

## Discussion

This study found some evidence that parental warmth and control are associated with children’s cognitive development. Children whose parent’s demonstrated low warmth at age 24 to 47 months had a 0.52-point lower IQ at age 8 than children whose parents demonstrated high warmth. The study also found that high controlling parenting was associated with 2.21-point lower IQ than less controlling parenting. Effect sizes of parenting practices were small (warmth: 0.03 standard deviation; control: 0.15 standard deviation) but may have an important impact at a population level [35]. It has been suggested that parental warmth influences children’s IQ by providing more support in problem solving, more engagement in positive parent-child interaction, and increased likelihood to encourage exploration and task persistence [52]. Meanwhile, high controlling parenting may have restricted the child’s ability to make autonomous choices, and impeded the child’s free expression of feeling and thinking, which in turn hinders their cognitive development.

The results of the effect-measure modification analyses provide some evidence to suggest that the associations between parenting practices (warmth and control) on childhood IQ differ according to temperament, although results need to be interpreted cautiously because the confidence intervals were wide in the strata of difficult temperament due to smaller numbers of children. We hypothesized that the associations between low parental warmth or high control and IQ would be more prominent among children with a difficult temperament, but there was almost no evidence of an exacerbated risk of lower IQ in this stratum. Instead, low warmth and high control parenting was associated with higher risks of low IQ among children with easy and other temperaments. This was surprising as previous research has suggested that children with easy temperament might be more adaptable or less susceptible to parenting practices [53,54]. Compared with children who have easy temperaments, there was a small 12–18% increased risk of lower IQ among children with a difficult temperament and therefore it is important that children with difficult temperaments are supported to realize their full cognitive potential. This might require different types of parenting support and this may be the subject of further research.

For both parenting warmth and control, results on effect-measure modification showed that there was no evidence that parenting interventions should be targeted to children with difficult temperament. Although we found that children with easy or other temperament may be more susceptible to low warmth or high control parenting than children with difficult temperament, children with easy temperament comprise a much larger proportion of the population (85%). As such, rather than targeting children with specific temperament, it may be more appropriate to provide parenting interventions as a universal program. While there is some evidence of effect-measure modification by temperament on the risk-ratio scale, it is difficult to determine the applicability of effect-measure modification on the risk-ratio scale for this research question.

This study has several advantages over previously published research on the association between parenting, temperament and IQ. First, we were able to make better causal inferences by adjusting for a wider range of potential confounders than have been used in many previous studies [55,56] especially when studying the effect-measure modification. However, it is possible that the results of this longitudinal cohort study remain open to residual and unmeasured confounding, as with all cohort studies. Second, the differential effect of parenting on IQ by child temperament was examined based on a strict definition of effect-measure modification. Other studies [56,57] have not differentiated effect-measure modification from interaction, and results are often not interpreted correctly. Third, assessing effect-measure modification on both the risk-difference and risk-ratio scale is transparent and provides information for readers to draw conclusions about effect-measure modification and the implications of the results. To our knowledge, this is the first study that has investigated the effect-measure modification by temperament on the association between parenting and IQ on both risk-difference and risk-ratio scales. Fourth, data were from a population-based prospective longitudinal study with a large sample, and we used multiple imputation to address potential bias due to missing data. However, several study limitations should also be noted. While the sample was representative of the population in the United Kingdom, the study sample was not very culturally diverse and since parenting styles may vary across cultures, we cannot generalize the results of this study to other ethnic groups or cultures. Future studies with more diverse cultural groups are required. In addition, although we have a large sample size (n = 7044), the wide confidence intervals from the effect-measure modification may be influenced by the small number of children with difficult temperament which reduces the power to detect effect-measure modification. It is also possible that children’s IQ might affect parenting, however we are unable to examine this due to the temporal order in which data were collected.

In summary, this study showed small effect sizes of parenting warmth and control at age 24 to 47 months on children’s IQ at age 8. We found no increased risk of low IQ as a result of parental warmth or control in temperamentally difficult children. As such, to improve children’s IQ, it may be more appropriate to offer interventions to improve parental warmth and decrease parental control as a universal program, rather than targeting to parents who have children with difficult temperament.

## Supporting Information

S1 File Appendix A. Effect-measure modification. Appendix B. Pearson’s correlation coefficients. Appendix C. Graphical presentation of results on effect-measure modification. Appendix D. Supporting information tables for effect-measure modification using different IQ cut-offs.
(DOCX)

S2 File Temperament questionnaire (PDF)
