# Supplementary material for: Parenting Practices at 24 to 47 Months and IQ at Age 8: Effect-Measure Modification by Infant Temperament
Source: PLoS One. 2016 Mar 30;11(3):e0152452. doi: 10.1371/journal.pone.0152452 (PMC4814065; doi:10.1371/journal.pone.0152452)
Supplement: S1 File — Appendix A. Effect-measure modification. Appendix B. Pearson’s correlation coefficients. Appendix C. Graphical presentation of results on effect-measure modification. Appendix D. Supporting information tables for effect-measure modification using different IQ cut-offs. (DOCX) [file pone.0152452.s001.docx]

**S1 File**

**Appendix A: Effect-measure modification**

The presence or absence and the magnitude of effect-measure modification depend on which scale the association is measured - risk-difference or risk-ratio scale.[[1](#_ENREF_1)] The effect-measure modification on the risk-difference scale, the relative excess risk due to interaction (RERI) is calculated as:[[2](#_ENREF_2),[3](#_ENREF_3)]

$$RERI={RR}_{P_{1}T_{1}}- {RR}_{P_{0}T_{1}}- {RR}_{P_{1}T_{0}}+ {RR}_{P_{0}T_{0}}$$

where *P* denotes parenting (0=high warmth; 1=low warmth) and *T* denotes temperament (0=easy/other; 1=difficult). RERI = 0 means no effect-measure modification; RERI > 0 means positive effect-measure modification; RERI< 0 means negative effect-measure modification.

The effect-measure modification on the risk-ratio scale, ratio of RRs is taken as:

$$\frac{{RR}_{P_{1}T_{1}}{RR}_{P_{0}T_{0}}}{{RR}_{P_{0}T_{1}}{RR}_{{P_{1}T}_{0}}}$$

Ratio of RRs = 1 means no effect-measure modification; ratio of RRs > 1 means positive effect-measure modification; ratio of RRs < 1 means negative effect-measure modification.

Examples 1 to 3 in the following were taken from Vanderweele [[3](#_ENREF_3)] to illustrate the scale dependence of effect-measure modification and the public health significance of the risk-difference scale.

1. An example of when effect-measure modification occurs on the risk-difference but not risk-ratio scale

|  | No alcohol | Alcohol |
| --- | --- | --- |
| Non-smokers | 0.02 | 0.05 |
| Smokers | 0.04 | 0.10 |

**Risk-difference scale:** 0.10-0.05-0.04+0.02 = 0.03

**Risk-ratio scale:** (0.10 x 0.02) / (0.05 x 0.04) = 1

1. An example of when effect-measure modification occurs on the risk-ratio but not risk-difference scale

|  | No alcohol | Alcohol |
| --- | --- | --- |
| Non-smokers | 0.02 | 0.05 |
| Smokers | 0.07 | 0.10 |

**Risk-difference scale:** 0.10-0.05-0.07+0.02 = 0

**Risk-ratio scale:** (0.10 x 0.02) / (0.05 x 0.07) = 0.57

1. An example of effect-measure modification on the risk-difference scale for public health interest

|  | No drug | Drug | Risk-difference | Risk-ratio |
| --- | --- | --- | --- | --- |
| Genotype A | 20 | 10 | 20-10=10 | 20/10=2 |
| Genotype B | 3 | 1 | 3-1=2 | 3/1=3 |

The risk-difference allows us to see the absolute gain in outcome if an intervention is targeted at certain subgroup. From the data above, the effect of drug on the risk-difference scale amongst individuals with genotype A is greater than the risk-difference amongst individuals with genotype B (10 vs 2). If we had 100 doses of the drug, we can improve the outcome in 10 additional individuals if the drug is given to people with genotype A. If the drug is given to all people with genotype B, we can improve outcome in 2 additional individuals. We would want to give the drugs to people with genotype A. The risk-ratio scale, however, may indicate the wrong subgroup to be targeted for intervention. As we can see from the data above, the risk-ratio is larger for subgroup with genotype B than for subgroup with genotype A (2 vs 3).

1. An example of effect-measure modification on the risk-ratio scale for investigation of disease etiology

|  | No asbestos | Asbestos |
| --- | --- | --- |
| Non-smokers | 1 | 3 |
| Smokers | 10 | 80 |

From the data above, there is evidence of effect-measure modification on the risk ratio scale [80 / (10 x 3) = 2.67]. This suggests that there may be a multiplicative relation between smoking and asbestos exposure in relation to lung cancer risk [[4](#_ENREF_4)]. One possible explanation may be that that smoking damages the cilia in the airways and thereby opens up for exposure from contaminated particles. However, it has been pointed out there is potential danger to use statistical interaction to draw conclusion about biological interaction [[5](#_ENREF_5),[6](#_ENREF_6)].

**Appendix B: Pearson’s correlation coefficients^a^**

|  | **Warmth** | **Control** | **Temperament** | **IQ** | **Birthweight** | **Gestational age** | **sex** | **Ethnicity** | **Maternal age** | **Maternal smoking** | **Maternal alcohol consumption** |
| --- | --- | --- | --- | --- | --- | --- | --- | --- | --- | --- | --- |
| **Warmth** | 1.00 |  |  |  |  |  |  |  |  |  |  |
| **Control** | 0.08 | 1.00 |  |  |  |  |  |  |  |  |  |
| **temperament** | 0.03 | -0.03 | 1.00 |  |  |  |  |  |  |  |  |
| **IQ** | -0.09 | -0.10 | -0.02 | 1.00 |  |  |  |  |  |  |  |
| **Birthweight** | -0.03 | 0.01 | -0.04 | 0.11 | 1.00 |  |  |  |  |  |  |
| **Gestational age** | -0.02 | -0.04 | -0.02 | 0.04 | 0.57 | 1.00 |  |  |  |  |  |
| **sex** | -0.08 | -0.19 | 0.04 | -0.00 | -0.09 | 0.05 | 1.00 |  |  |  |  |
| **Ethnicity** | 0.00 | -0.01 | 0.06 | -0.04 | -0.08 | -0.04 | -0.00 | 1.00 |  |  |  |
| **Maternal age** | -0.18 | 0.02 | -0.02 | 0.21 | 0.08 | -0.01 | -0.02 | -0.05 | 1.00 |  |  |
| **Maternal smoking** | 0.07 | -0.01 | 0.01 | -0.14 | -0.14 | -0.01 | -0.02 | 0.03 | -0.22 | 1.00 |  |
| **Maternal alcohol consumption** | 0.03 | -0.00 | 0.01 | 0.01 | -0.00 | 0.01 | -0.01 | -0.03 | 0.11 | 0.12 | 1.00 |
| **Partner status** | 0.03 | -0.01 | 0.04 | -0.12 | -0.06 | -0.00 | -0.00 | 0.17 | -0.24 | 0.20 | 0.07 |
| **Home ownership** | 0.05 | 0.00 | 0.05 | -0.21 | -0.08 | -0.02 | -0.00 | 0.13 | -0.34 | 0.30 | -0.00 |
| **Household crowding** | 0.01 | 0.02 | 0.04 | -0.14 | -0.02 | 0.01 | -0.01 | 0.08 | -0.15 | 0.15 | 0.00 |
| **Maternal education** | -0.14 | -0.09 | 0.01 | 0.38 | 0.06 | 0.02 | 0.01 | -0.01 | 0.31 | -0.24 | 0.04 |
| **Partner’s education** | -0.11 | -0.05 | -0.02 | 0.37 | 0.07 | 0.02 | -0.01 | -0.04 | 0.32 | -0.24 | 0.03 |
| **Parental social class** | 0.09 | 0.05 | 0.02 | -0.27 | 0.05 | -0.00 | 0.00 | 0.03 | -0.29 | 0.20 | -0.03 |
| **Financial difficulties** | 0.06 | -0.01 | 0.08 | -0.13 | -0.04 | 0.00 | -0.00 | 0.07 | -0.08 | 0.15 | 0.01 |
| **Social support** | -0.05 | -0.02 | -0.12 | 0.11 | 0.01 | 0.02 | 0.01 | -0.08 | 0.06 | -0.08 | -0.07 |
| **Maternal depression** | 0.08 | -0.01 | 0.11 | -0.12 | -0.07 | -0.05 | -0.00 | 0.08 | -0.14 | 0.18 | 0.02 |
| **Partner’s depression** | 0.05 | -0.01 | 0.06 | -0.09 | -0.04 | -0.04 | -0.00 | 0.10 | -0.10 | 0.13 | 0.04 |
| **Maternal health** | 0.03 | -0.01 | 0.06 | 0.00 | -0.02 | -0.02 | -0.00 | 0.01 | 0.01 | 0.05 | 0.02 |

|  | **Partner status** | **Home ownership** | **Household crowding** | **Maternal education** | **Partner’s education** | **Parental social class** | **Financial difficulties** | **Social support** | **Maternal depression** | **Partner’s depression** | **Maternal health** |
| --- | --- | --- | --- | --- | --- | --- | --- | --- | --- | --- | --- |
| **Partner status** | 1.00 |  |  |  |  |  |  |  |  |  |  |
| **Home ownership** | 0.29 | 1.00 |  |  |  |  |  |  |  |  |  |
| **Household crowding** | 0.12 | 0.30 | 1.00 |  |  |  |  |  |  |  |  |
| **Maternal education** | -0.15 | -0.26 | -0.16 | 1.00 |  |  |  |  |  |  |  |
| **Partner’s education** | -0.19 | -0.29 | -0.16 | 0.56 | 1.00 |  |  |  |  |  |  |
| **Parental social class** | 0.19 | 0.31 | 0.18 | -0.45 | -0.44 | 1.00 |  |  |  |  |  |
| **Financial difficulties** | 0.12 | 0.16 | 0.10 | -0.12 | -0.16 | 0.14 | 1.00 |  |  |  |  |
| **Social support** | -0.12 | -0.15 | -0.09 | 0.08 | 0.12 | -0.09 | -0.14 | 1.00 |  |  |  |
| **Maternal depression** | 0.15 | 0.19 | 0.14 | -0.11 | -0.11 | 0.11 | 0.21 | -0.29 | 1.00 |  |  |
| **Partner’s depression** | 0.19 | 0.18 | 0.12 | -0.09 | -0.12 | 0.10 | 0.13 | -0.17 | 0.22 | 1.00 |  |
| **Maternal health** | 0.02 | 0.01 | 0.03 | 0.00 | -0.00 | -0.01 | 0.08 | -0.09 | 0.18 | 0.07 | 1.00 |

^a^ Correlation coefficients need to be interpreted carefully because all variables are categorical, except IQ, birthweight, gestational age, and maternal age.

**Appendix C: Graphical presentation of results on effect-measure modification**

We used the same information from Table 4 in the manuscript to present effect-measure modification in a graph (S1 Figure 1). Compared with children with high warmth and easy temperament (reference category, RR=1.00), children with low warmth and easy temperament had 14% increased risk of having low IQ; children with high warmth and difficult temperament had 17% increased risk of having low IQ; and children with low warmth and difficult temperament had 12% increased risk of having low IQ.

Estimates in S1 Figure 2 were taken from Table 5 in our manuscript. Compared with children with low control and easy temperament (reference category, RR=1.00), children with high control and easy temperament had 31% increased risk of having low IQ; children with low control and difficult temperament had 18% increased risk of having low IQ; and children with high control and difficult temperament had 18% increased risk of having low IQ.

The RERI was calculated as the difference in estimates between these four groups. The ratio of RRs was calculated as the ratio of estimates between these four groups.

**Appendix D: Supporting information tables for effect-measure modification using different IQ cut-offs**

**Effect-measure modification of the effect of parenting warmth on IQ by child temperament (Imputed sample, n = 7044)**

1. **IQ (<80)**

|  | High warmth parenting | | Low warmth parenting | | RR (95% CI) for low warmth parenting within strata of temperament type |
| --- | --- | --- | --- | --- | --- |
|  | N Low IQ/High IQ | RR (95% CI) | N Low IQ/High IQ | RR (95% CI) |  |
| Easy or other temperament | 151/2697 | 1.00 (Ref) | 259/2923 | 1.26 (1.02, 1.57), p=0.032 | 1.23 (0.98, 1.53), p=0.073 |
| Difficult temperament | 28/410 | 1.33 (0.89, 1.99), p=0.158 | 57/519 | 1.43 (1.04, 1.97), p=0.030 | 1.07 (0.65, 1.75), p=0.788 |

RERI= -0.17 (-0.83, 0.50), p=0.619

Ratio of RRs= 0.85 (0.52, 1.39), p=0.514

1. **IQ (<90)**

|  | High warmth parenting | | Low warmth parenting | | RR (95% CI) for low warmth parenting within strata of temperament type |
| --- | --- | --- | --- | --- | --- |
|  | N Low IQ/High IQ | RR (95% CI) | N Low IQ/High IQ | RR (95% CI) |  |
| Easy or other temperament | 469/2379 | 1.00 (Ref) | 663/2519 | 1.07 (0.95, 1.22), p=0.263 | 1.05 (0.93, 1.19), p=0.473 |
| Difficult temperament | 71/367 | 1.09 (0.85, 1.40), p=0.504 | 133/443 | 1.08 (0.88, 1.33), p=0.465 | 1.00 (0.73, 1.39), p=0.973 |

RERI= -0.08 (-0.43, 0.27), p=0.642

Ratio of RRs= 0.92 (0.67, 1.28), p=0.628

RRs are adjusted for parental control, maternal smoking, alcohol consumption, birth weight, gestation at birth, sex, ethnicity, maternal age, partner status, financial difficulties, maternal and partner’s education, parental social class, home ownership, household crowding, maternal health, social support, maternal and partner’s depression

**Effect-measure modification of the effect of parenting control on IQ by child temperament (Imputed sample, n = 7044)**

1. **IQ (<80)**

|  | Less controlling parenting | | High controlling parenting | | RR (95% CI) for high control parenting within strata of temperament type |
| --- | --- | --- | --- | --- | --- |
|  | N Low IQ/High IQ | RR (95% CI) | N Low IQ/High IQ | RR (95% CI) |  |
| Easy or other temperament | 194/3350 | 1.00 (Ref) | 216/2270 | 1.34 (1.09, 1.65), p=0.005 | 1.32 (1.06, 1.64), p=0.012 |
| Difficult temperament | 47/581 | 1.31 (0.94, 1.83), p=0.112 | 38/348 | 1.48 (1.01, 2.08), p=0.043 | 1.13 (0.69, 1.84), p=0.623 |

RERI=-0.19 (-0.87, 0.47), p=0.560

Ratio of RRs= 0.83 (0.51, 1.35), p=0.446

1. **IQ (<90)**

|  | Less controlling parenting | | High controlling parenting | | RR (95% CI) for high control parenting within strata of temperament type |
| --- | --- | --- | --- | --- | --- |
|  | N Low IQ/High IQ | RR (95% CI) | N Low IQ/High IQ | RR (95% CI) |  |
| Easy or other temperament | 580/2964 | 1.00 (Ref) | 552/1934 | 1.21 (1.08, 1.38), p=0.002 | 1.22 (1.08, 1.39), p=0.002 |
| Difficult temperament | 116/512 | 1.12 (0.91, 1.38), p=0.300 | 88/298 | 1.14 (0.90, 1.48), p=0.264 | 1.02 (0.74, 1.41), p=0.883 |

RERI= -0.19 (-0.55, 0.17), p=0.311

Ratio of RRs= 0.84 (0.61, 1.16), p=0.293

RRs are adjusted for parental warmth, maternal smoking, alcohol consumption, birth weight, gestation at birth, sex, ethnicity, maternal age, partner status, financial difficulties, maternal and partner’s education, parental social class, home ownership, household crowding, maternal health, social support, maternal and partner’s depression

**References**

1. Rothman K, Greenland S, Walker A. Concepts of interaction. Am J Epidemiol. 1980;112: 467-470.

2. Knol MJ, VanderWeele TJ. Recommendations for presenting analyses of effect modification and interaction. Int J Epidemiol. 2012;41: 514-520.

3. VanderWeele TJ, Knol MJ. A tutorial on interaction. Epidemiol Methods. 2014;3: 1-40.

4. Ahlbom A, Alfredson L. Interaction: A word with two meanings create confusion. Eur J Epidemiol. 2005;20: 563-564.

5. Siemiatycki J, Thomas D. Biological models and statistical interactions: An example from multistage carcinogenesis. Int J Epidemiol. 1981;10: 383-387.

6. Greenland S, Lash T, Rothman K. Concepts of interaction. In: Rothman K, Greenland S, Lash T, editors. Modern epidemiology. 3^rd^ ed. Philadelphia: Lippincott, Williams & Wilkins; 2012. pp. 71-83.
