# Supplementary material for: Parenting Practices at 24 to 47 Months and IQ at Age 8: Effect-Measure Modification by Infant Temperament
Source: PLoS One. 2016 Mar 30;11(3):e0152452. doi: 10.1371/journal.pone.0152452 (PMC4814065; doi:10.1371/journal.pone.0152452)
Supplement: S2 File — (PDF) [file pone.0152452.s002.pdf]

S2 File:Temperament questionnaire

These questions are about how your baby behaves. Although some of them seem similar to one another, please answer them all. How often has the baby's recent behaviour been like the following descriptions:

		Almost never	Rarely	Usually does not	Usually does	Often	Almost always
H1.	She eats about the same amount of solid food (within 2 spoon-fuls) from day to day	1	2	3	4	5	6
H2.	She is fussy on waking up and going to sleep (frowns, cries)						
H3.	She plays with a toy for less than a minute and then looks for another toy or activity						
H4.	She sits still while watching TV or other nearby activity (such as children playing)						
H5.	She accepts straight away a change in place or position of feeding or person doing it						
H6.	She accepts nail cutting without protest						
H7.	Her hunger cry can be stopped for more than a minute by picking up, putting on a bib, or giving a dummy						
H8.	She plays continuously for more than 10 minutes with a favourite toy						
H9.	She accepts her bath any time of the day without resisting it						
H10.	She takes feeding quietly with mild expressions of likes and dislikes						
H11.	She indicates discomfort (fussy/squirms) when she has a dirty nappy						
H12.	She lies quietly in the bath						
H13.	She wants and takes milk feedings at about the same time (within one hour) from day to day						
H14.	She is shy (turns away or clings to you) on meeting another child for the first time						
H15.	She continues to fuss when her nappy is changed despite efforts to distract her with game, toy or singing etc.						
H16.	She amuses herself for half an hour or more in her cot or playpen (looking at mobile, playing with toy)						
H17.	She moves about a lot (kicks, grabs, squirms) during nappy change and dressing						
H18.	She vigorously resists additional food or milk when full (spits out, clamps mouth closed, pushes spoon away etc)						

	Almost never	Rarely	Usually does not	Usually does	Often	Almost always
H19. She resists changes in feeding schedule (1 hour or more) even after 2 tries	1	2	3	4	5	6
H20. Her bowel movements come at different times from day to day (over 1 hour difference)						
H21. She stops play and watches if someone walks by						
H22. She ignores voices or other ordinary sounds when playing with a favourite toy						
H23. She makes happy sounds (coos, laughs) when having her nappy changed, or being dressed						
H24. She accepts new foods straight away, swallowing them promptly						
H25. She watches other children playing for less than a minute and then looks elsewhere						
H26. She reacts mildly (just blinks or is startled briefly) to a bright light such as flash bulb or sunlight let in by drawing back curtain						
H27. She is pleasant (smiles, laughs) when first arriving in unfamiliar places (friend's house, shop)						
H28. She gets sleepy at about the same time each evening (within half hour)						
H29. She accepts regular procedures (hair brushing, face washing, etc) at any time without protest						
H30. She perseveres for many minutes when working on a new skill (rolling over, picking up object, etc)						
H31. She moves a lot (squirms, bounces, kicks) while lying awake in her cot						
H32. She objects to being bathed in a different place or by a different person even after 2 or 3 tries						
H33. For the first few minutes in a new place or situation (new shop or home) she is fretful						
H34. She notices, looks carefully at changes in your appearance or dress (hairdo, unfamiliar clothing)						
H35. She reacts strongly to foods, whether positively (smacks lips, laughs, squeals) or negatively (cries)						
H36. She is pleasant (coos, smiles,						

		Almost never	Rarely	Usually does not	Usually does	Often	Almost always
	etc) during procedures like hair brushing or face washing						
H37.	She continues to cry in spite of several minutes of soothing	1	2	3	4	5	6
H38.	She keeps trying to get a desired toy, which is out of reach for 2 minutes or more						
H39.	She greets a new toy with a loud voice and much expression of feeling (whether positive or negative)						
H40.	She plays actively with her parents - much movement of arms, legs, body						
H41.	She watches another toy when offered even though already holding one						
H42.	At home her initial reaction to strangers is acceptance						
H43.	She wants daytime naps at differing times (over 1 hour difference) from day to day						
H44.	She continues eating solid foods without reacting to differences in taste or consistency						
H45.	She cries when left to play alone						
H46.	She adjusts within 10 mins to new surroundings (home, shop, play area)						
H47.	Her naps are about the same length from day to day						
H48.	She moves about much during feeding (squirms, kicks, grabs)						
H49.	She reacts (stares or is startled) to sudden changes in lighting (flash bulbs, turning on light)						
H50.	She can be soothed by talking or games when sleepy						
H51.	She displays much feeling (vigorous laughing or crying) during nappy change or dressing						
H52.	She lies still when asleep and wakes up in the same position						
H53.	She reacts to changes in her milk (type or temperature) or if given juice instead						
H54.	She can be calmed for a few minutes by being picked up and played with, if fussing about a dirty nappy						
H55.	She wants and takes solid food at about the same time (within 1 hour) from day to						

day

		Almost never	Rarely	Usually does not	Usually does	Often	Almost always
H56.	She is content (smiles, coos) during interruptions of milk or solid feeding	1	2	3	4	5	6
H57.	She accepts within a few minutes a change in place of bath or person giving it						
H58.	She cries for less than 1 minute when given an injection						
H59.	She shows much bodily movement (kicks, waves, arms) when given an injection						
H60.	She continues to react to a loud noise (hammering, barking dog, etc) heard several times in the same day						
H61.	Her initial reaction is withdrawal (turns head, spits out) when consistency, flavour or temperature of solid foods is changed						
H62.	Her time of waking in the morning varies greatly (by 1 hour or more from day to day)						
H63.	She continues to reject disliked food or medicine in spite of your efforts to distract with games or tricks						
H64.	She reacts even to a gentle touch (is startled, wriggles, laughs, cries)						
H65.	She reacts strongly to strangers: laughing or crying						
H66.	She actively grasps or touches objects within her reach (hair, spoon, glasses, etc)						
H67.	She will take any food offered without seeming to notice the difference						
H68.	Her period of greatest physical activity comes at the same time every day						
H69.	She appears bothered (cries, squirms) when first put down in a different sleeping place						
H70.	She reacts mildly to meeting familiar people (quiet smiles or no response)						
H71.	She wants an extra feed at a different time each day (over 1 hour difference)						
H72.	She is still wary or frightened of strangers after 15 mins						
H73.	She lies still and moves little while playing with toys						

		Almost never	Rarely	Usually does not	Usually does	Often	Almost always
H74.	She can be distracted from fussing or squirming during a procedure (nail cutting, hair brushing, etc) by a game, singing, TV, etc	1	2	3	4	5	6
H75.	She remains pleasant or calm with minor injuries (bumps, pinches)						
H76.	Her initial reaction to seeing doctor is acceptance (smiles, coos)						
H77.	She reacts to a disliked food even if it is mixed with a preferred one						
H78.	She plays quietly and calmly with toys (little vocal or other noises)						
H79.	She lies still during procedures like hair brushing or nail cutting						
H80.	She stops sucking and looks when she hears an unusual noise (telephone, door bell) when drinking milk						
H81.	She pays attention to a game with a parent for only a minute or so						
H82.	She is calm in the bath. Like or dislike is mildly expressed (smiles or frowns)						
H83.	She requires introduction of a new food on 3 or more occasions before she will accept (swallow) it						
H84.	Her first reaction to any new procedure (first haircut, new medicine, etc) is objection						
H85.	She acts the same when the nappy is wet as when it is dry						
H86.	She is fussy or cries during a physical examination by a doctor						
H87.	She accepts changes in solid foods (type, amount, timing) within 1 or 2 tries						
H88.	She moves much and for several minutes or more when playing by herself (kicking, waving arms and bouncing)						
